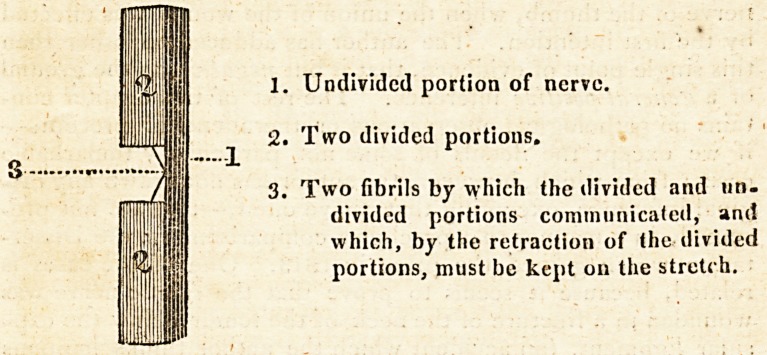# Book Reviews

**Published:** 1821-04

**Authors:** 


### CRITICAL ANALYSES

OF

### RECENT PUBLICATIONS, IN THE DIFFERENT BRANCHES OF MEDICINE AND SURGERY.

"I would have men know, that, though I reprehend the easie passing over of the causes of things "by ascribing them to secret and hidden vertues and properties; (for this hath arrested and laid "asleepe all true enquiry and indications;) yet I doe not understand but that, in the practical "part of knowledge, much will be left to experience and probation, whereunto indication cannot "so fully reach: and this not only in *specie*, but in *individuo*. Yet it was well said, *Vere scire* "*esse per causas scire*."—BACON.

*History and Method of Cure of the various Species of Palsy: being the first Part of the second Volume of a Treatise on Nervous Diseases.* By JOHN COOKE, M.D. F.A.S.; Fellow of the Royal College of Physicians; and late Physician to the London Hospital. 8vo. pp. 215. Longman and Co. London, 1821.

THE intentions of the author in the publication of this series of Treatises on Nervous Diseases, were stated by us in the review of that on Apoplexy; and the judgment we then formed of the value of the work, has certainly not been considered too favourable on further consideration. We have ourselves found the preceding volume of much utility, for the purposes of practice as well as of pathological inquiry; and, to students especially, there cannot be a doubt but that it will prove eminently useful. The most important part of the work is the summary it furnishes of the best pathological principles and therapeutical precepts respecting the subjects of it, that prevail in the present day: but the historical view of those points presents matter by no means devoid of interest to those who desire to contribute to the improvement of medicine. The history of this science displays a region far too extensive for the investigation of persons of ordinary habits of inquiry, and unless a man is acquainted with what has already been done and thought, he is likely, if he attempts to attain any thing that is new, to employ himself in researches which have already produced every thing of which they are capable; to frame hypotheses which have already been entertained, and overthrown by the opposition of facts of which he is ignorant; or, at much expense of time and intellectual labour, to arrive at conclusions which he might have seen demonstrated in a few words by some extant author. Had (for example) some of our modern physiologists known merely what is stated in the writings of GALEN, they could have saved years of fruitless labour that might have been otherwise advantageously exerted. It is not, however, a mere train of unconnected notions, such as constitute the articles in the generality of *Dictionaries*, and even a considerable proportion of those of one of such pretensions as the *Dictionaire des Sciences Medicales;* it is a perspicuous and methodic, or (to use a metaphoric expression,) a digested, account of the facts and opinions promulgated in former times that is qualified to be of the utility above indicated: there is as much diversity in the value of the two, as well as in the talents requisite for their construction, as there is between such a use of extensive erudition as is manifested in the works of KAIMS, and the quotation of at least a score of authors in every page, without which the writers of a continental nation think their books would not make a respectable appearance, any more than their professors would without their chains and ermine. The application of those remarks will be perceived on a reference to the works under consideration. The present volume is, however, even better constructed than the preceding one: there is in it a greater proportion of original reflection; and a more abstract character is given to the citations, by which the conciseness of the work is increased, whilst its utility is not lessened.

In the first chapter the author treats of the "Definition, Distinction, and general History," of Palsy. He shows that the ancients very generally considered apoplexy and palsy as diseases of the same nature, but different in degree: apoplexy being an universal palsy, or palsy a partial apoplexy; and hence, as we stated in our review of the preceding Treatise, HIPPOCRATES speaks of apoplexy in the leg, &c. After having cited the definitions of palsy given by ARETÆUS, BOERHAAVE, CULLEN, YOUNG, and GOOD, Dr. Cooke proposes the following one. "It is a disease in which there is a diminution, or an entire loss, of the power of voluntary motion, or of sensation, or of both, in some particular part or parts of the body, without coma." A concise account is next adduced of the precursory phenomena, and then the symptoms are thus detailed in a general way.

"Palsy chiefly consists in the loss of the power of voluntary motion, for sensation, in a greater or less degree, generally remains; nay, in certain cases it is morbidly increased. I have seen several instances in which paralytic persons have felt very violent pain in the parts affected, particularly in the shoulder and arm. These remarks might be confirmed by quotations from various authors. I never saw a case of palsy in which sensation was entirely lost; and an eminent physician of great experience asserts that a total loss of feeling in this disease is extremely rare. The other senses are often but little injured; sometimes they remain wholly unimpaired, and several instances might be adduced in which they appeared to be preternaturally acute. Dr. Heberden attended a paralytic person, whose sense of smelling became so exquisite as to furnish perpetual occasions of disgust and uneasiness; and he mentions one case in which *all* the senses became exceedingly acute.

"The vital and natural functions in palsy are generally but little affected. The actions of the heart and lungs are indeed sometimes more languid, and the secretions and excretions less regular than in a state of health; but this is not usually the case."

It has generally been considered that a diminution of temperature of the affected parts, is a necessary consequence of paralysis; and it has been supposed that this effect has arisen from less heat being developed in them from want of due nervous influence. Another explanation of this phenomenon has been proposed by Dr. Abercrombie: he is inclined to believe that paralytic parts do not necessarily become colder than natural, but that the variation of temperature in them arises from their having lost the power of regulating their temperature; so that they *cool* when exposed to a temperature less than that of the body, and *become heated* when exposed to one above it, more readily than healthy parts. He says, according to Dr. Cooke's statement, that he had long ago observed that paralytic limbs are sometimes warmer than sound limbs, but without being able to account for it; and he relates, in support of his views of this subject, an instance in which "a medical gentleman, on visiting a paralytic patient, was astonished to find the paralytic arm so intensely hot that he could not touch it. He was at first very much surprised, but found, upon inquiry, that the patient had, by the advice of a friend, applied to the arm a quantity of very hot bran, or something of that kind very hot, which had been removed a short time before his visit."

Dr. Cooke remarks on this point, that, "if Dr. Abercrombie's notion be correct, that paralytic limbs lose the power of preserving their temperature; or, in other words, if their power of resisting changes of temperature be lost; it appears to me that the temperature of such parts would be less than that of other parts exposed to a medium of heat inferior to that of the human body; which is always the case in temperate climates."

The other remarkable phenomena in the affected parts, are the wasting of them, and the sense of formication often experienced. After having discussed these points, Dr. Cooke adverts to the depression, imbecility, and sometimes almost annihilation, of the intellect; and he refers to several of the curious cases on record, in which the memory especially has been defective. Some persons have, indeed, not preserved the consciousness of self-identity; and it is a curious circumstance that the forgetfulness has especially existed in regard to nouns substantive and the knowledge of languages. In many cases of this kind, the patients have only been unable to pronounce the words, whilst they have remembered the names of things, and could, in some instances, repeat the letters of a name distinctly. This was the case with Professor Broussonnet: he could not pronounce the name of his daughter, after an attack of palsy; but he could articulate, in proper order, the letters of which it was constituted.

Dr. Cooke says, "In cases of persons recovering from palsy, I have often observed that the parts most distant from the head are first restored to sense and motion. In hemiplegia, it almost always happens that the power of the leg returns long before that of the arm. I have even seen more than one case, in which the arm of the affected side has remained paralytic for several years after the restoration of the leg." Similar remarks have been made by others, and the facts they indicate are worthy of the consideration of physiologists. The restoration of the power of sense and motion is, we believe, evinced in the same way in paraplegia: it was so in the case related in the last Number of this Journal, by Dr. Venturi.

A very interesting fact, relating to this subject, has just been mentioned to us by Dr. Harrison. A boy had had nearly complete paralysis of one leg for nine years, from a curvature of the lumbar portion of the spine: as far as related to motion, he could only throw the limb a little backwards, and had not the least power of motion of the toes. The distortion of the spine was almost wholly removed immediately, by one operation, and the boy could *instantly* afterwards move his toes. He is gradually regaining the power of motion in the muscles of the limb generally.

Dr. Cooke treats, in the next instance, of the *distinction* of paralysis: he adopts that of Cullen, into hemiplegia, paraplegia, and partialis. He thinks the addition of the species *venenata* objectionable, as well as the others founded, agreeably to the system of Sauvages, on supposed causes of the disease.

The second chapter treats expressly of the history of Hemiplegia. Paulus Ægineta, Dr. Cooke says, seems to have been the first who applied this term to paralysis affecting one side of the body. It is, he continues, "in a great proportion of cases, preceded by an apoplectic fit, which is sometimes so slight and transient as to have escaped general notice; but the attentive observer will almost always perceive certain symptoms indicative of the stroke,—particularly distortion of the muscles of the mouth, drowsiness, forgetfulness, and dullness of apprehension, in a greater or less degree." The stomach and bowels have been generally supposed to be in a torpid state in this disease, chiefly from their apparent insensibility to the agency of medicines, and the costiveness which accompanies the disease; but Dr. Cooke remarks that this torpor may be merely apparent, and that "it would appear, from the experiments of Magendie, and the observations of other physicians, who have found inflammation of the intestinal tube produced by ordinary purgatives under these circumstances, that the stomach and bowels are, in fact, particularly irritable; vomiting and purging not taking place from such medicines, merely because the action of the muscles, necessarily for those functions, cannot be excited." These phenomena are more frequently obvious in apoplexy than in hemiplegia simply: it is but rarely that the abdominal muscles are not excited to action, in the latter affection, by irritation of the bowels. Retention of urine in apoplexy, is often witnessed from the same causes. It is true that involuntary evacuations of the feces and urine often happen in paraplegia, and sometimes in apoplexy: but here the sphincters of the rectum and bladder have lost their powers, and the contraction of the intestines and bladder, respectively, are then sufficient alone to effect the expulsion of their contents, which is not the case when the sphincters exert their ordinary functions: here the concurrence of action of voluntary muscles is requisite.

HOFFMANN, from having seen enlargement of the liver with hemiplegia of the right side, hypothetically inferred that the viscera of the diseased side are disposed to become affected because they borrow so many branches from the external nerves. The influence of the cerebral or spinal nerves on secretion or the circulation,—it does not seem easy to determine which,—is shown in a remarkable manner by a case cited by MORGAGNI, (from the *Ephem. Nat. Curios.* c. 3, obs. 64:) it was that of an old man who was affected with palsy in the right side, and at the same time with jaundice; the jaundice being confined to the paralytic side so accurately, that even the right part of the nose was yellow, whilst the left retained its natural colour. Morgagni, although his alternatives in the way of explanation are generally so numerous, could only remark on this curious case,—"*quando idem flavum sanguinis serum non minus per sinistrum latus, quam per dexterum; nisi forte credas, per laxiores hujus fibras lentius promotum magis inficere potuisse.*"

The palsy which follows apoplexy, Dr. Cooke says, is generally a complete hemiplegia: but there are many anomalies in this disease, some of the most remarkable of which are adduced. FABRICIUS speaks of palsy in one arm and in the foot of the opposite side; and RAMAZZINI, HEISTER, and SENAC, of loss of feeling only in one leg, and loss of motion only in the other. Sauvages enumerates, amongst the species of hemiplegia, one which he denominates intermittens: "It is that hemiplegia," he observes, "which comes on every day, and, after some hours, recedes with an accession of quotidian fever." Loss of sense only, and of motion only, in individual instances, are not very rare. Dr. Cooke concludes his citations of this kind with a citation, from the seventh volume of the Medico-Chirurgical Transactions, of the cases of De Saussaure and Vieusseux; that related by Mr. Keratry, (which was inserted in a late Number of this Journal;) and an extraordinary case that occurred to his own observation.

"An officer of high rank in the army, who is now about sixty years of age, was, in the year 1795, affected with a diminution of power in the right hand. This complaint increased, notwithstanding a variety of modes of treatment, till the year 1800; when, after a course of mercury, recommended by Mr. Cline, its further progress was stopped, since which time the disease has remained stationary. The peculiar circumstances of this case are the following: The muscles of the left arm, from the shoulder to the elbow, are much wasted, and greatly diminished in power; while the muscles of the fore-arm are not at all diminished in size, and but little in power. The state of the right side is just the reverse: the muscles of the upper arm being of their natural size, and possessing their full power; whilst those of the fore-arm are very much wasted, and their motion, especially that of the fingers, almost entirely abolished. In all other respects, this gentleman appears to be perfectly well. No cause for this disease can be assigned; nor did any method of treatment afford the smallest relief, till the mercurial course was adopted, when the progress of the disorder was arrested in the year above mentioned. Since that time no attempts to remove this complaint have been made, yet it does not increase."

After having described the ordinary immediate consequences and final termination of hemiplegia, the author treats of Paraplegia. Van Swieten remarks that it was the custom, in his time, in the medical schools, to call that disease paraplegia, in which voluntary motion ceases in all parts below the neck. Dr. Good applies the term to paralysis "of the lower half of the body on both sides;" and it seems to have been very commonly used in this sense by modern writers. It is but rarely that cases corresponding with the definition alluded to by Van Swieten, are really witnessed; for an injury seated so high in the spinal marrow as to produce such an affection, generally rapidly destroys life, by its influence on the function of respiration. In one case which we witnessed, where the spinal marrow had been much injured about the junction of the second and third cervical vertebræ, by a fall, the patient lived two days, dilating his chest, for inspiration, only by the intercostal, scaleni, and other muscles attached to its superior part; the diaphragm being, apparently, quiescent. Dr. Cooke mentions the accuracy of the knowledge of Galen, of the influence of lesions in various parts of the spinal marrow on the rest of the body, and the proofs he gives of his acquaintance with paraplegia from disease originally of the parts constituting the spinal column.

Some erroneous views seem to have been taken of the seat of the cause of paraplegia, from disease of the brain having been found in cases where it has existed, without its having been considered that the real cause of the paraplegia might have been in the spinal marrow, whilst the cerebral disease was merely a casual contingency; as Dr. Harrison argues in a late paper inserted in this Journal. This chapter terminates with a concise account of a case of that remarkable form of disease referred to by SAUVAGES, in his Nosology, article *Scelotyrbe festinans.* The case above alluded to first appeared as an occasional paroxysm of an inability to walk slowly or to stand still, though, if the patient set out and run, he could proceed to some distance without falling to the ground. There was evidently no affection of the brain in this case; for there was no vertigo or disturbance of the senses, and the power of motion in the arms was not diminished; so that, when the fits came on, (which they usually did after the patient, a man forty-five years of age, had walked for two or three miles,) in situations where the patient could grasp with his hands any thing sufficiently firm, he could support himself upright. When they occurred in such a place as an open field, he used to quicken his pace gradually to the most rapid walk, then to that of running, which became quicker and quicker until he arrived at some resting-place, or, if this were distant above three or four hundred yards, until he fell to the ground. A sense of weight in the lower limbs succeeded these paroxysms, and they had been preceded, for two or three years, by uneasy feelings about the loins and sacrum, leading the patient to suppose he had hemorrhoids, though no evidence of them could be perceived on examination. The case degenerated into a constant diminution of the power of motion, and, at the end of four years, purulent matter formed about the sacrum; the paraplegia became complete, and the patient soon afterwards died.

CUVIER seems to have explained the most remarkable phenomena proper to this affection, when he says that "several quadrupeds, with whom standing on two feet is very difficult, can nevertheless walk thus for a certain length of time with tolerable facility, because, in general, the act of walking is much less difficult than that of station; the same muscles, in the former, not being in so constant a state of contraction; and because it is easier to correct the vacillations by other contrary and alternative vacillations, as may be done in walking, than to prevent them entirely." We find, too, that a drunken man can keep on his feet whilst he is staggering forwards, though he would fall down if he were to attempt to stand still.

The fourth chapter treats of Partial Paralysis, that is, palsy "which affects less than half the body, or some one particular part or organ." This species includes, in conformity with the author's definition of palsy, diseases of organs that consist in a loss either of sensation or of motion only,—as paralytic affections of the nerves of sight, hearing, &c. and want of power of motion in the eyes, loss of speech, &c.

Cases of paralysis of one or more muscles in various other parts of the body, without any obvious lesion from which they may originate, are not unfrequently witnessed. After having noticed these varieties of this species of disease, Dr. Cooke enters into a discussion of the question how it happens that sensation and the power of motion are not both lost, if one of them be lost, "since they both depend upon the nerves?" More modern physiologists have done nothing more than adopt the explanation of Erasistratus and Galen on this point, which Dr. Cooke seems to think, himself, is the most plausible one that has hitherto been proposed.

The fifth chapter treats of the Causes of Palsy. "The chief *predisposing* causes of general palsy," the author says, "are those which I have enumerated and explained in the first volume of this work, as the predisposing causes of apoplexy; such as advanced age, hereditary feeble constitution, and especially a leucophlegmatic, pituitous, or dropsical habit." The *exciting* causes of palsy also resemble those of apoplexy. After having discussed those points, the author examines the hypothesis of Dr. Serres, contained in his paper in the *Annuaire Medico-Chirurgicale.* Dr. Cooke thinks that "the conclusions which he [Dr. Serres] has drawn from his reasoning, are too general, and by no means strictly logical;" and he shows that they are "controverted by facts which lead to positive conclusions, not to negative conclusions," as those of Dr. Serres are. We need not follow the author through his examinations of this subject, and his discussions respecting the seats of the causes of paraplegia and partial paralysis.

The subject next noticed is the circumstance that the palsy in hemiplegia generally affects the opposite side to that in which the apparent cause of it exists in the brain. On this point, too, Dr. Serres evinces his injudicious generalization, and asserts that the paralysis is always on the opposite side to that of the disease in the brain. Of the incorrectness of this assertion, we had the most satisfactory proof a few days since. A girl, eleven years of age, who had been a patient of the Welbeck-street Dispensary, under the care of Dr. Outram, had paralysis on the right side only, for six weeks, when she came under our care. Her death took place at the end of about four weeks longer. Two large cavities, containing coagulated blood, considerably altered in its appearance, were found in the right hemisphere of the cerebrum; whilst the left hemisphere was devoid of the slightest appearance of disease.

It has generally been found, in paralysis from disease of the spinal marrow, that the palsy has been on the same side as that affected, when the medulla has been injured on one side only; but that this, too, is not universally the case, is shown by a case quoted from PORTAL by the author.

The explanations given of the phenomena above alluded to, in hemiplegia, by ARETÆUS, (that it arises from a crossing of the nerves at their origin;) LANCISI, (from a decussation of the fibres in the corpus callosum;) SOEMMERING and HALLER, (from a crossing of the fibres of the brain immediately below the origin of the lingual nerves;) Dr. YELLOLY, (who seems to think that SANTORINI is most correct, in considering that the supposed decussation is in the tuberculum annulare, whilst he refutes the inference of GALL on this point;) are passed in review; and the author concludes his considerations on this subject by remarking, that "notwithstanding the observations and reasonings of anatomists and physiologists on this subject, much obscurity remains; yet, on the whole, I think it seems more probable that a decussation of nerves takes place in the tuberculum annulare than in any other part. If the minute structure of the brain were better developed,—if it could be shown to consist of converging fibres,—we might better understand how injuries done to one side of the brain, especially in the higher parts of the hemispheres, might produce palsy on the opposite side of the body; but, though such a fibrous structure of the brain has been supposed to have been seen by Leuwenhoek, Bidloo, Cowper, Gall and Spurzheim, and others, its existence has not been satisfactorily proved."

The sixth chapter treats of Dissections, Diagnosis, and Prognosis. This is constituted, as regards the first subject, of accounts of the observations of BONETUS, LIEUTAUD, WILLIS, MORGAGNI, Dr. ABERCROMBIE, Dr. SERRES, ROUCHOUX, RIOBE, and PORTAL. The only novel observations adduced are those of Mr. CHARLES BELL, who says, in relation to the alterations that take place in nerves in parts affected with palsy, that "nerves, if not employed, degenerate into a sort of cellular membrane."

After passing in review the remarks of former authors respecting the prognosis, Dr. Cooke says,

"As far as my own experience enables me to judge, the prognosis in the general palsies must be almost always unfavourable. I have seen many cases of recovery from palsy in a very considerable degree; but I do not recollect more than one or two cases, of a complete restoration, both of sensation and motion, in the whole of the side of a person who had been affected with a perfect hemiplegia. When this species of palsy depends upon an injury done to one side of the brain which is almost always the case, I am inclined to think that the mischief is seldom, if ever, entirely obliterated, and the disease wholly removed. On the dissection of persons after palsy, either evident disease is found in the brain, or marks of the existence of former disease, which had given occasion to the complaint; and, although Messrs. Rochoux and Riobe have adduced good reasons for believing that fluids effused have been absorbed, and that cavities in the brain have been sometimes closed, yet the mischief may not have been completely removed, nor the brain perfectly restored to its healthy state; and, whilst any morbid cause capable of producing palsy continues in any degree to exist, it is natural to suppose that palsy in some degree would remain. Reasoning from appearances after death from palsy, would lead us to conclude that the disease almost always, in a greater or less degree, does remain. Instances may, no doubt, be adduced of perfect recovery from palsy; but I am persuaded that such are of very rare occurrence. If persons affected with hemiplegia do not become apoplectic in a short time, it often happens that, after a certain degree of amelioration, the disease becomes stationary, or very gradually proceeds, even for several years, before it terminates fatally."

The seventh, and last, chapter is on the Treatment of Palsy. Dr. Cooke first discusses that which should be employed in hemiplegia, which, he again remarks here, "is, in a very great proportion of cases, the consequence of apoplexy: therefore, the plan to be adopted, both for the prevention and the cure of the former of these diseases, is very much like that recommended for the latter; indeed, it differs chiefly in degree." In conformity, however, with his plan, by which each treatise is rendered distinct and complete in itself, the author discusses here the measures to be adopted as prophylactics, and for the treatment of the actual disease under particular consideration. The similarity in the modes of treatment is, however, considered only as precisely proper for hemiplegia in its early state. "When the disease has subsisted for a length of time; when the apoplectic symptoms have disappeared; when plethora, or marks of determination of blood to the head, are no longer present, our mode of proceeding should be different; and certain remedies may be prescribed, which, under other circumstances, would be dangerous. These remedies are chiefly stimulants externally applied, or internally taken." Besides the numerous physical excitants we possess, moral impressions have been occasionally employed by physicians; and there are numerous instances recorded of their efficacy, when those of the former class had failed: but, Dr. Cooke says, we derive no practical advantage from a knowledge of these facts, "the excitement of the passions not being sufficiently under our management and control."

"Of the stimulants to be applied externally, there perhaps is none more safe and efficacious than friction by the hand or by the flesh-brush. I have, in several instances, seen very beneficial effects from a long perseverance in the use of this simple remedy. Friction may be rendered more powerful by stimulating liniments, of which we have many different kinds, such as the fossil acids and volatile alkalies, combined with oil or lard, with a view of rendering them less acrid and corrosive; essential and distilled oils; preparations from resins, gum-resins, &c. Among the most powerful external applications for the purpose of restoring action and sensation, we may reckon blisters and sinapisms, especially the latter, which are amongst the most powerful rubefacients that we can employ. Blisters and sinapisms are very generally recommended in palsy, because they are considered safe and efficacious. One of the Greek physicians, however, very properly says, that, when parts are entirely deprived of sensation and motion, we ought to be careful that sinapisms do not operate too much; the patient, through loss of feeling, not being able to judge of their effects.* Some practitioners are in the habit of applying blisters, or other stimulants, to the head, immediately after the accession of hemiplegia; but I am of opinion that we ought not to make such applications in plethoric constitutions, and especially when the disease is the consequence of apoplexy, till some blood has been taken away; and I think that, when such stimulants are used, they should be applied on that side of the head which is opposite to the paralytic side; because anatomists have ascertained, as above mentioned, that, in a very great proportion of instances, the cause of hemiplegia is seated in some part of the brain opposite to the side affected. Celsus recommends, in these cases, the application of nettles to the surface of the part affected, and also mustard."†

After noticing the statements that have been made respecting warm and cold bathing, Dr. Cooke says, "On this subject I cannot give an opinion from my own experience, but, on the whole, the observations which others have made would lead me to prefer, in palsy, the application of warmth by bathing to that of cold; as the former is more under our command than the latter. If cold does not produce re-action, or if it give occasion to a very great re-action, it would, probably, do mischief." The use of electricity, with the evidences for its efficacy, is next discussed. Dr. Cooke himself says, that, from his own observations of the effects of electricity in paralytic affections, he ventures to recommend it, with due precautions respecting the cases to which it is applied, and the mode in which it is employed. He says,

"Applied in a certain manner, electricity is a most powerful stimu-

* Paul Ægineta, lib. iii. c. 13.

† Prodest etiam torpentis membri summam cutem exasperâsse, vel urticis cæsam, vel imposito sinapi, sic ut ubi rubere cæperit corpus, hæc removeantur.—Celsus, lib. iii. c. 27.

lant to the nervous system, and therefore much has been expected from it in the cure of palsy; but, as it is also a stimulus to the sanguiferous system, it has often been hurtful in those palsies which depend upon a compression of the brain, and especially when it has been so employed as to act upon the vessels of the head. It is only to be considered safe when its operation is confined to parts somewhat remote from the head; and as, when very strongly administered, it is capable of destroying the mobility of the nervous power, it should be used with only moderate force. Advantage is to be expected rather from a repetition of it than from its force; and it seems particularly suited to the cure of those palsies which have been produced by the application of narcotic powers.* Where electricity has been prejudicial, it has probably been too violently applied, and no greater force should be used than that which may be sufficient to remove or alleviate disease: thus, shocks should never be used when a cure may be effected by sparks; sparks should be avoided when the required effect can be obtained by the wooden point; and if the metal point be thought sufficient, it should be preferred."

When employed in conformity with those precepts, he does not recollect a single instance in which it appeared to do mischief. Dr. Bardsley expresses himself strongly in favour of the employment of galvanism in paralysis, and he relates some well-marked and decisive instances of the successful application of it in various forms of this disease.

"In the application of galvanism in these cases, Dr. Bardsley recommends the method employed by Mr. Wilkinson: for instance, in a case of hemiplegia of the right side, accompanied by vertigo, loss of memory, and involuntary discharge of urine, he began with half a dozen plates, of two inches and a quarter square, and applied the conducting wires in such a manner as to direct the galvanic influence through the brain. The sensation was powerful and unpleasant; but, by degress, the patient was able to bear the power of a dozen plates. The galvanic fluid was likewise directed along the spine and the upper and lower extremities, in as powerful a degree as the patient's feelings would admit. In about a fortnight, this person became entirely free from any appearance of disease, except a slight retraction of the muscles of the face, which was not attended with pain or any inconvenience.†

"For a minute account of the cases of palsy under the care of Dr. Bardsley, treated by galvanism, particularly of one, most singular and deplorable, to which he calls our attention, as furnishing an unequivocal testimony in favour of the practice, I must refer to his work. Dr. Bardsley draws from his experiments the following general conclusions: 1. That galvanism, judiciously administered, is a safe and powerful remedy in most paralytic diseases. 2. That, as far as three comparative trials will allow an inference, the efficacy of galvanism in

* Cullen. † Bardsley's Medical Sketches, p. 186, 187.

paralysis is superior to that of electricity. 3. That galvanism agrees with electricity in its sensible effects upon the body. 4. That, when the brain is required to form part of the circle, the galvanic influence ought to be very cautiously administered. 5. If no sensible advantage accrue from a steady and properly-regulated application of this remedy, after a trial of a week or ten days, in paralytic affections, especially where the brain is operated upon, its use ought to be laid aside. 6. When the pulse has become quicker and firmer; the local, as well as general, temperature of the body increased; the feelings, both mental and corporeal, somewhat enlivened; and the altered secretions better regulated; it is proper to infer, from such indications, that galvanism may be persisted in with a fair prospect of ultimate success. 7. Where both sensibility and irritability are so greatly exhausted as not to render the patient susceptible of the galvanic stimulus by the ordinary means; or where, from the unusual thickness of the cuticle, it forms a barrier to the transmission of the fluid, it will be necessary to excoriate the surface by blistering ointment, and apply the metallic points to the raw skin; but the pain and agitation frequently induced by administering the remedy through so sensible a medium, must be guarded against, by adapting the number of plates to the increased degree of sensibility. Dr. Bardsley states, that the galvanic stimulus is an efficacious, though not certain, remedy in paralytic affections; and he is induced to think that, in all cases which appear to originate solely from a diminished excitement in the sensorium, galvanism is to be preferred to electricity."

Similar inferences respecting the superiority of the efficacy of galvanism to that of electricity, have been drawn by Mr. La Beaume, whose opportunities for comparing the results of the use of the two measures have been very extensive. Galvanism, too, can be applied with more precision than electricity, from the regulation of its power being more nicely and completely manageable.

The actual cautery was much employed by the ancients in palsy, and is now much used by the French, in the way of applications of moxa. On noticing an instance of the efficacy of this remedy, Dr. Cooke says, "might not a discharge from the spine, produced by other means, have been equally successful in this case? Was not the application of the moxa preferred on account of the quickness of its operation?" From our personal observations, we are disposed to think that the moxa is superior in its efficacy, in certain cases, to every other mode of cautery. The moral impressions attending its application—from the patient seeing, or knowing, that he has a fire burning on a part of his body, that he is expecting every instant to arrive at his skin,—are often very powerful; and these, perhaps, have somewhat to do with the efficacy of the remedy.

Of the internal remedies of a stimulant kind, the most celebrated are the rhus toxicodendron, nux vomica, horse-radish, and mustard-seeds. Of the first, Dr. Cooke says, "The cases adduced by Dr. Alderson, illustrating the good effects of the employment of the rhus toxicodendron in hemiplegia, are very striking, and afford encouragement to a trial of it in those cases of palsy where the employment of stimulants is indicated." It is hardly necessary to inculcate caution in the use of this remedy, as it is the most actively poisonous plant of a species that comprises several deleterious substances. The nux vomica is a variety of the most poisonous species *(strychnos)* of known vegetables. Dr. Cooke gives an abstract of the recorded cases in which it has proved efficacious, subsequently to the publication of the paper of Dr. Fouquier on this subject; but we need not cite them in a particular manner, as an account of the most remarkable of them has already been inserted in this Journal. Dr. Cooke does not appear to have himself employed either this remedy or the arnica. He says, he thinks he has seen cantharides useful in several cases: of camphor, ether, lavender, valerian, castor, and "other medicines called nervine," he cannot speak from experience.

The treatment of *paraplegia* from disease in the spine, is next noticed. Dr. Cooke only mentions the practice of Mr. Pott, and the objections that have been made to the use of issues. We shall soon have to present the readers of this Journal with something very interesting on this subject from Dr. Harrison. The new view he has taken of the origin of the disease, has led to a mode of treatment that has been attended with very extraordinary success: spinal distortions, under his practice, are, indeed, amongst the most easily manageable and curable of all diseases of any severity of character.

From the subject just mentioned, the author proceeds to speak of the treatment of *partial palsies*, and in the first place of amaurosis. The only novel remarks on this subject, (with the exception of some statements respecting the influence of electricity, by Mr. Partington, more favourable to the use of that remedy than those of the generality of practitioners,) are taken from Mr. Travers's Synopsis of the Diseases of the Eye. The precision of observation and the nice distinctions of disease and practical indications, manifest in that work, render those remarks particularly interesting: we shall, therefore, transcribe Dr. Cooke's abstract on this subject.

"Mr. Travers thinks that the treatment of amaurosis should be almost exclusively constitutional. He places no confidence in external applications,—such as stimulant vapours, drops, and ointments; spirituous and aromatic embrocations, sternutories, &c. He makes an exception, however, in favour of cupping, issues, or setons, in certain cases, and of blisters in almost all. He never witnessed any advantage in this disorder from the employment of electricity or galvanism: he has not known any real benefit from what are called antispasmodic and anti-nervous medicines; nor from the exhibition of emetics, though, from respect to authority, he has fairly tried them in many instances.

"In most cases of amaurosis, Mr. Travers depends on the regulation of the visceral functions, and the employment of such restoratives as the system requires, and can bear. The blue pill, with colocynth, rhubarb, and aloes, and the combination of soda with rhubarb and calumba or gentian, are best adapted, he thinks, to the former purpose. The exhibition of general tonics, he says, is often indicated; and he has seen much benefit from the mineral acids, bark, steel, and arsenic when admissible, after a due regulation of the digestive functions. In recent and sudden amaurosis, Mr. Travers recommends a mild administration of mercury, but salivation, he thinks, is always hurtful; and he is of opinion that 'all cases of direct debility, and proper paralysis of the retina, are aggravated by the loss of blood.'"

Cases of partial palsy more commonly come under the care of surgeons than physicians; and hence it is, probably, that this part of the work presents a view of the medical measures that have been proposed by others, with but few original observations or practical inferences by the author.

An abstract of a report by Dr. Gordon from the minutes of the Army Medical Board, respecting the occurrence of apoplexy and palsy in the army, is attached to the work as an appendix. Dr. Cooke says, it appears to him (from the inspection of the table of returns for a period of six months,) "that the proportion of cases of apoplexy and palsy, as they occur in the army, is very small; a circumstance which may, perhaps, be explained by observing that soldiers generally quit a military life before they arrive at the age when these disorders most frequently occur; and that those who are strongly predisposed to them are, as Dr. Gordon has observed, on that account refused admission into the army."

"Dr. Gordon," he adds, "does not find that any particular make or conformation of body was observable in those soldiers who were affected with apoplexy and palsy. The chief exciting causes of these disorders were intoxication, exposure to the rays of the sun, drinking cold water, and bathing in cold water when the body was heated. Dr. Gordon remarks, that apoplexy often followed epilepsy, long continued fevers, and visceral disease, especially dysentery; and that serous apoplexy sometimes came on after a species of marasmus, denominated *cachexia Africana;* sometimes after an improper use of mercury; and frequently after blows and falls from horseback.

"The apoplectic seizure, in this climate, chiefly occurs between the ages of thirty and fifty; but in warm climates it takes place without much reference to any particular age, as it arises chiefly from exposure to the sun, and the abuse of spirituous liquors. The appearances found after death much resemble those which I have at large described.

"Dr. Gordon observes, that, in the treatment of this disease in the army, the remedies almost wholly relied upon were bleeding, both general and topical, including arteriotomy; the application of blisters, and the administration of cathartic medicines."

---

*A Dissertation on the Treatment of Morbid Local Affections of Nerves: to which the Jacksonian Prize was adjudged by the Royal College of Surgeons.* By Joseph Swan, Member of the Royal College of Surgeons, and Surgeon to the Lincoln County Hospital. 8vo. pp. 196. J. Drury, London. 1820.

"Non scribo hoc temere. Quo minus familiaris sum, hoc sum ad investigandum curiosior."
Cic. *Ep. ad Fam.* lib. iv. Ep. xiii.

THE dearth of knowledge on the subject of this dissertation, and the urgency of the reasons medical practitioners have for desiring that their information should be more extensive; as well as the hopes entertained that late researches have contributed to supply the deficiency; were strongly expressed by the Court of Assistants of the College of Surgeons, when they proposed the question on which it is founded, only seven years after this question had given origin to one of the best monographies in medical literature. It is true that the dissertation before us relates especially to the *treatment* of diseases of the nerves; whilst the other, just alluded to, comprises, with the same object, considerations on the physiological and pathological relations of this part of the human economy: but, such men as those who proposed the question, could not have contemplated the probability of any important improvement in the treatment of diseases of organs so extensively and variously related in the system as the nerves, without a corresponding improvement of our knowledge of their functions and morbid affections; or that therapeutical precepts could be applied with any considerable degree of confidence and precision, if unconnected with particular pathological indications. Under these circumstances, the work to which the stipulated premium has been conceded, appears with claims on the attention of medical men of a very forcible kind; and it is especially incumbent on those who profess to view with a critical regard the progress of medicine, to examine it with strict severity, and endeavour to determine the precise extent of the original knowledge it is qualified to impart. These remarks are intended to serve as an apology for, perhaps, a more rigid censorial review of this work than may, as we are disposed to believe, be most beneficially applied to the generality of books; for, it is better that some few inanities, plagiarisms, and amusing absurdities, should pass current for valuable, as well as rightful, property of their promulgators, than that so dreary and disheartening a picture should be drawn of the history of medical science, as must be done were the real state of its present relative perfection to that of former and remote ages, displayed in an unreserved manner.

Although it is, in the title-page, expressly stated that this dissertation relates to the *treatment* of diseases of the nerves, the author, in conformity with a sentiment expressed above, does not merely discuss therapeutical indications and the means for fulfilling them: he endeavours, also, to establish some inferences respecting the nature of those diseases.

Another preliminary remark which we have to make, is that, as it appears, the author has considered it incumbent on him to give a view of all that is known respecting the means proper for the treatment of "local" diseases of the nerves; and hence it is, as we infer, that some of his discussions do not contain any thing that is profferred as original.

There is nothing remarkable in the author's Preface, excepting the following statement: "When a part has been deprived of the nervous influence, by its communication with the sensorium being intercepted, the functions of the part to which the nerve is distributed are suspended, and are incapable of being reproduced until the divided portions of nerve has become reunited, except through electricity."

We shall have occasion to notice the evidence which has been adduced in proof of this assertion, when this point becomes the subject of discussion in the course of this review.

The dissertation commences with the consideration of "Diseases and Injuries of the Nerves in general." The author thinks it prudent to treat of those diseases "under two distinct heads; viz. those that affect the nerves belonging to the senses, and those that affect such nerves as are under the influence of the will."—"A third division," he continues, "might be added, which would include the ganglian system, belonging chiefly to the grand sympathetic nerve, and distributed in great measure to the thoracic and abdominal viscera; but, as I do not know that any particular researches have been made by pathologists into this part of the nervous system, to ascertain whether disease occasions any change in it to take place in the parts to which it is distributed,* and as it is not much connected with

* There is, it appears, some error (to be attributed to the printer, perhaps,) in the distribution of the words of part of this sentence; but the author's meaning seems to be obvious.

the department of the surgeon, I shall pass it over; for, as I could say very little more than what is theoretical, it would not answer the intention for which the present subject was proposed."

The author, in conformity with the view above indicated, treats, in the first instance, of diseases of the nerves of the senses; and he commences with "diseases and injuries of the olfactory nerves." On this subject he remarks that

"The power of the nerves constituting the sense of smell, may be diminished or destroyed by the frequent application of strong odours to the nose, or from an inflammation of the Schneiderian membrane. The same thing may likewise happen from pressure on the origins of the nerves by hydatids, or an accumulation of water in the lateral ventricles of the brain, or from their being involved in a diseased action going on at the under surface of the anterior lobes of the brain, or from a diminution of the foramina of the cribriform plate of the ethmoid bone. When there is an inflammation of the Schneiderian membrane, which takes away this sense, leeches may be applied to the outside of the nose; and the inside may be anointed, by means of a feather, with some cooling ointment; and purging medicines may be given. All the other diseases are generally beyond the reach of art."

These remarks are followed by an account of a case in which, the author says, "the sense of smell of the right nostril appeared to have been suspended by an inflammatory action going on about the crista galli." The author's inference here is very plausible—from the evidence furnished by the symptoms and seat of pain—but, as the case did not terminate in death, (but in restoration of the function,) or so as to admit of an examination of the parts, the inference is merely plausible. The author refers to a case in Morgagni (Epist. ix. art. 25,) where there was proof of inflammation in the seat alluded to accompanying similar symptoms.

The author then remarks that the functions of the olfactory nerves are sometimes so diseased as to produce a sense of unpleasant odours; effects analogous to the sensations of flashes of light and sounds, without the proper external excitants, from diseases of the optic and auditory nerves, respectively.

This is all that is advanced respecting diseases of the olfactory nerves; and, but for the practice we intend to adopt in this article, it would be superfluous to remark that it does not contain any thing that is novel.

"Diseases and injuries of the Optic Nerve," are next considered. The author says, (we quote the remarks, because we do not mean to omit to notice any of his reflections.)

"Diseases and injuries of this nerve, and its expansion the retina, are almost always attended with a destruction of its functions; so that, though every other tunic of the eye and its humours are perfectly sound, and capable of transmitting freely the rays of light, no impression is made by them [the *rays of light* the author must mean, not the *tunics and humours of the eye*, as the grammatical construction of the passage indicates,] on the retina, which constitutes the disease termed amaurosis."

After the foregoing passage, there follows an account of some of the causes and the principal symptoms of amaurosis, as it generally takes place. This account contains nothing that is new, nor is it characterized by any remarkable degree of precision. It is succeeded by some therapeutical precepts, of a general and common-place character, and devoid of originality.

The next subjects of discussion are "Diseases and injuries of the Gustatory Nerves." The author remarks, that "the gustatory nerves are sometimes injured by being violently bruised between the teeth; and, though there is no apparent injury of the tongue, those powers of the nerve, producing the sense of taste, will be destroyed. The evidence adduced in proof of this statement, is a case related by Sir EVERARD HOME, and published in the Philosophical Transactions.* In addition to this, Mr. Swan remarks that morbid states of the tongue occur, in which things, at other times sapid, will make no impression on that organ during such a condition of it, or the impressions will be very different from what they ordinarily are; or there will be a sense of various unpleasant tastes in the mouth, without the presence of the proper external causes.

Respecting "diseases and injuries of the Auditory Nerves," the author relates a case of deafness of one ear from a supposed fracture of some part of the base of the skull; which deafness still existed at the end of "some months" from the time of the receipt of the injury.

An account follows of the symptoms of the ordinary cases of deafness, and some remarks respecting their treatment, which are equally devoid of novelty with the parts of the work previously noticed. After this, we arrive at a disquisition relative to hearing that is of a physiological character, and which the author says contains "something new on this subject." This is a transcription of a paper published in the Medico-Chirurgical Transactions,† with the addition of the history of a case serving to show that a sense of articulate sounds may be perceived by means of impressions on the nerves of the face. But Mr. Swan's notions in this respect are not so precisely novel as he imagines: the probability of the connexion of the facial nerves with the sense of hearing, had been stated by MAGENDIE, in his Elements of Physiology, (tom. i. p. 100;)

* An abstract of it is given in the eleventh volume of this Journal.

† It was transcribed in the 250th Number of this Journal.

and many curious facts tending to show that such a faculty as that supposed to be possessed by the facial nerves particularly, is also occasionally possessed by the nerves of touch in general, were published, several years since, by PFINGSTEN.* Mr. Swan thinks it probable that, by exercise and proper instruments, the powers of the facial nerves might be so much developed, that children, otherwise deaf, might be made to hear tolerably well by this medium.

The "diseases and injuries of the nerves of Touch," are so much the same, the author says, as those of the nerves with which they are connected, that he shall consider them altogether.

The second chapter treats of "Diseases and Injuries of the Nerves of Voluntary Motion, &c. in general." The author here commences by remarking, that

"In paralytic affections, the nerves of voluntary motion are generally those that suffer; and, though the nerves of feeling, or those of the skin, generally arise and are chiefly connected with them, they do not appear to suffer in the same degree that those do which are destined to supply the muscles. As, for example, in a paralytic limb pain and itching will be complained of, and the sense of feeling will remain at the time the muscles have not the power of obeying the will."

Sensation, it may be added, is sometimes abolished whilst the power of voluntary motion remains. These facts have been considered to favour strongly the notion of the nerves of sensation being throughout distinct from the nerves serving for motion: but this notion is opposed by the apparent intimate confusion of all the nerves of a limb in the plexuses. Mr. Swan is disposed to adopt the same notion that GALEN also thought most plausible, that "the muscles of voluntary motion require the nerves to be in the most perfect state to enable them to act; and that a less degree of perfection is necessary for them to perform the functions required for the sense of feeling." In support of this view he observes, that, in a great degree of pressure on the spinal marrow, there is generally a loss both of sensation and the power of voluntary motion; whilst, in less degrees of pressure, the faculty of sense generally remains to a certain extent: and, alluding to the existence of the power of motion without feeling, he says,

"Upon due consideration of the subject, we cannot, I think, be much surprised that, when the nerves have been divided or injured, as in paralysis, a great difference should exist as to the parts to which they are distributed requiring different degrees of perfection in their

---

* *Vieljaehrige Erfahrung über die Gehoerfehler der Saubstummen.* Kiel, 1802. An account of some of Pfingsten's experiments was given in an abstract of a dissertation by Professor ROSENTHAL, in the 256th Number of this Journal.

restoration, to enable them to perform their respective functions; for the parts to which the nerves producing the sense of feeling are distributed, are to be acted upon mechanically by things external to, and unconnected with, the animal; whilst those intended to serve the purposes of voluntary motion, are to be produced by a nicer stimulus, —viz. through the agency of the will; which is something so subtle as not to be entirely comprehended by us, either as to the manner in which it is formed by the brain, or how it is communicated by the brain to the parts it calls into action.

"Though it is sometimes the case, yet it is, as I have just now stated, a comparatively rare occurrence for the nerves of sensation to suffer from paralysis; and those of voluntary motion, though arising from the same trunks, to be but little affected by it. When it does occur, I conceive that such an alteration takes place in the skin, or the parts composing it, as to prevent the proper exercise of the functions of the nerves distributed to it."*

There is something more in this matter than is here regarded, for here are no indications for an explanation of the existence of insensibility, not in the skin merely, but throughout the whole of a limb, whilst the power of voluntary motion was perfect.†. A little reflection will suggest an abundance of conjectures on this subject; but, as our senses here can make us acquainted only with effects, it is not probable that we shall ever arrive at a knowledge of the causes of the phenomena alluded to. We may suppose that the want of the power of motion may depend on alterations in the properties of the muscles themselves, with or without alterations of those of the nerves; or that (which is plausible) voluntary motion depends on the transmission of some influence from remote parts, which transmission to the limb may be interrupted; whilst the transmission of influence from it, concerned in sensation, may be undisturbed;‡ or the faculties of sense and the power of voluntary motion may be the effects of distinct properties of the

* "This affection most commonly arises from a disorder of the digestive organs; and, if the brain is affected at the same time, it suffers from the same cause. As far as I have seen, when paralysis arises from pressure on the brain or medulla spinalis, the voluntary nerves always suffer with those of feeling; and I do not see how it can be otherwise."

† See vol. xlii. p 297, of this Journal, for a remarkable example; and *l'Histoire de l'Academie des Sciences*, 1743.

‡ There is a curious fact related, with different views, by CUVIER, respecting the probable existence of a sense of *feeling* without the presence of the brain, that, as it appears to us, has not been regarded with the attention it seems to merit. Cuvier (in an experiment to determine the functions of the inferior glottis of birds,) cut off the head and neck of a duck: "The animal afterwards walked a few steps; and, *when it was struck, it uttered several cries*, which, although weaker than those it gave when it had its head, were nevertheless very sensible." —*Leçons d'Anatomie Comp.* tom. iv. p. 454.

Many analogous statements, though less precisely applicable in the views with which this account is cited, have been made by men whose veracity and accuracy of observation cannot well be doubted; as will be seen on a reference to the physiological writings of Whytt, Perrault, Unzer, Bonnet, and Fontana. But, same structure; one species of which may maintain its ordinary relations with the brain, whilst the other is destroyed, or has these relations suspended. Some writers have attempted to explain this point, by stating that motion depends on an active state of the nerves, whilst these organs are *passive* in sensation; but those who can conceive the nerves to be passive in this function, have an imagination qualified very differently from ours. As, however, we can attain no sensible evidence of the causes of the phenomena, no conjecture respecting them can be any thing better than a more or less plausible supposition, founded on loose analogies, and must therefore be adapted for the amusement of our curiosity rather than the purposes of the practice of medicine. We revert to the dissertation before us, where our last transcript terminates.

Mr. Swan remarks, here, for the further support of the view he has adopted, the fact, which had already been noticed, that a certain state of circulation in the skin is necessary for the perfection of its sensation. He adduces, however, some original observations relative to this point, from a structure of the nostrils of the horse that appears to him to be formed for the perfection of the sense of smell.

"Beneath the Schneiderian membrane there are numerous sinuses, and many of them of considerable size, which have frequent communications with each other, and appear to be composed of a very thin and inelastic membrane, which is very strong, and perfectly smooth in the inside: within the sinuses are contained very delicate and extremely elastic vessels, which may be called veins, as they appear to be filled with venous blood; and, by their being thus situated within sinuses of a determinate size, they are capable of being distended to a certain degree only; which provision is necessary, as their extreme delicacy would otherwise either endanger their very frequent breaking from over-distension, or be the cause of much injury to the very delicate nerves, by a too-great pressure that would be thus made on them.

"This structure, I have no doubt, generally exists in animals, and may be very satisfactorily demonstrated in the horse; and it must, I think, appear to any one examining its peculiarities attentively, that it was not formed merely for returning the blood from the nose, but that it was made for distending the Schneiderian membrane, so as to give it a proper degree of tension to enable the nerves to receive more acutely the impressions from the odorous particles when applied to them; exactly in the same manner that it is required for the nerves of the penis to produce their peculiar sensations, that the parts connected with them should be properly distended with blood."

---

in the cases alluded to, what are called voluntary motions were performed: yet, as these motions are effected in some animals when deprived of brain and spinal marrow, it seems a necessary inference that they arise from causes different from those productive of our ordinary and proper *voluntary* motions.

Mr. Swan then advances the objections against the inference of a diversity in the nerves serving for sense and motion that have been often repeated; and with these the second chapter terminates.

The third chapter treats of "Diseases of the Nerves of Voluntary Motion," which are, the author says, of two kinds, active and passive.

"The active diseases are all those affections of the nerves attended by pain, and frequently by a motion of the affected part,—as tic douloureux, &c.

"The passive are those affections termed paralysis.

"In the active, at the part which appears to be the seat of the disease, there is an increased action of the blood-vessels, and likewise an increased heat; whilst in the passive, there is quite a contrary state.

"Those local complaints which appear to originate spontaneously, or in some cases where a slight wound has been inflicted, I believe to be only symptomatic of a general irritability of the brain and nervous system. The almost constant failure of topical remedies, and of the division of the affected nerve, must lead to the conclusion that the cause of the local diseased action, or primary affection, must reside in some other part of the body: and if we inquire into the causes of the local active affections of the nerves, it will be found that the atonic state of the body, or whatever tends to render the brain and nervous system irritable, will generally be found the most frequent."

These statements are either erroneous or, to our powers of comprehension, somewhat obscure. In the first place, "*active diseases*" of the nerves are not always attended by pain. A case is related in the Dissertation which gained the Jacksonian prize in 1813, of a tumor of the radial nerve, the formation of which had not been preceded by "pain or spasm." We do not, we must suppose, understand the author's meaning when he states that *active* diseases are frequently attended by a *motion of the affected part*. That there is always "an increased action of the blood-vessels, and likewise an increased heat," in "active" diseases of the nerves, is a supposition to which there are not wanting valid objections: the parts about the affected nerve in *tic douloureux* (an "active" disease, according to the author,) are not unfrequently colder than natural, and evidence of increased action of the blood-vessels is often wholly absent. It is now so general a custom, after having said all we know about the origin of a disease, to bring in inordinate *irritability* as the cause of all the rest, that the author can hardly be blamed for the vague remarks he has made on this point. But, in truth, this term irritability is merely a veil for ignorance that it would be more prudent to acknowledge: it is only a substitute for the *peccant humours* and *fermentations* of the older pathologists, and is, perhaps, not an advantageous one; for the latter do convey ideas of something positive, and therefore would still keep us in the way of observation and research; whilst the former, like all admissions of occult agents, is, as Bacon expresses, only qualified to arrest and lay asleep all true inquiry and indications. When we have some precise ideas of things, there is a chance, should they be erroneous, that we discover them to be so, and adopt correct ones in their place; but when we have no precise ideas, and yet cheat ourselves into a belief that we have, by the use of a *term*, there is no ground for expectations of improvement.

The author, after the remarks above alluded to, proceeds to say that women are more liable than men to this irritability, which itself will arise from undue mental exertions, certain passions, improper regimen, and disorder of the stomach. These general preliminaries on this point, then, contain nothing that is novel. The particular discussions commence with one on "painful affections of the Nerves of the Head and Face." "These complaints," the author says, "have been variously denominated,—intermitting pain of the head; hemicrania; tic douloureux, &c.; but they appear to be all the same disease, only varying in situation and degree." After describing the ordinary symptoms of this neuralgia, and stating that it is sometimes accompanied with increased action of the blood-vessels, the author says,—

"It appears to me that the irritation of the nerve is the cause of the increased action of the blood-vessels: nevertheless, this increased action may tend to increase or keep up the irritation of the nerve.

"It has frequently happened, after an operation in which a nerve has been principally concerned, that, either during the healing of the wound or after it has become completely cicatrized, if an increased action of the blood-vessels is produced, as is shown by inflammation about the part, the painful sensations resembling tic douloureux are produced. By this I would not say, that in this complaint [that is, we presume, tic douloureux,] there is an inflammation of the nerve, because I think other facts go to prove that there is not; but it shows that the increased heat and action have a decided effect in keeping up the complaint. The nerves may become enlarged from irritation, as in a case I shall relate, in the same way the muscles are from continued action: but when there has been inflammation of a nerve, though only of the chronic kind,—and to which sort that of tic douloureux must bear the greatest resemblance, if it were inflammation,—there would be the same change of structure that takes place in all continued inflammations of other parts of the body; viz. an enlargement from the deposit of coagulable lymph. This is shown when there has been a chronic inflammation of the extremities of the nerves in a stump, or when the nerves have been confined to a part that has been long subject to inflammation; and if this had been the case in tic douloureux, I think it would not have passed unnoticed."

These remarks are pretty good, as far as they extend, but they present only a faint and very partial glimpse of the admirable discussion on the same point, in the Dissertation which gained the prize in 1813. The author's remarks respecting the origin of this affection, are merely repetitions of common-place considerations. A case follows of tic douloureux, consequent on a blow over the right eye, which continued for ten weeks, and then suddenly disappeared on the occurrence of an eruption, like that of nettle-rash, all over the patient's body.

With respect to the treatment of tic douloureux, the author says, there appear to him to be two principal indications: "the first consists in strengthening the constitution, and thereby enabling it to counteract the habit which favours the continuance of the irritation; the second, in allaying the local irritation." The first is to be fulfilled by the exhibition of tonic remedies; the best of which, he thinks, is cinchona. This medicine has for some years been the favourite remedy of the French practitioners. Leeches, evaporating lotions, cold or warm fomentations, and an opiate liniment, are the measures for the second indication. A case is related, to prove the efficacy of these remedies. Another case is then adduced, that was also cured by the same means, where the disease ensued from a slight external injury. A third case, occurring subsequently to considerable disorder of the system, immediately consequent on the poisonous effects of verdigris, is afterwards related: it was treated, successfully, with cinchona. The author then alludes to the use of the other remedies which have been proposed, and amongst them the section of the affected nerves, without, however,—we regret to have such constant occasion for this repetition,—adding any original observations or inferences either of a general or particular kind; and we cannot say that what he does advance is characterized by any extraordinary degree of precision.

The fifth chapter treats of "painful Affections of various Nerves," in other parts of the body than the face: as instances of which, the following case is related; and two, of a similar kind, by Mr. Earle, (in the Medico-Chirurgical Transactions,) and Mr. Abernethy, (in his Surgical Observations,) are referred to.

"Mrs. W. had a pain in the left arm, which extended, in the course of the ulnar nerve, from the elbow to the little and ring fingers, both of which were weak and painful to the touch; the pain was not constant, but came on by fits. There was an evident disturbance of the digestive organs, with palpitations of the heart.

"She used a spirituous embrocation for the arm, and took five grains of the mercurial pill at bed-time, and a mixture with camphor and the volatile tincture of valerian; by which the pain was diminished. She was then attacked by a severe affection of the uterus; and after some time, when she was recovering from this complaint, the pain in the nerve ceased entirely, and never returned."

When a section of the nerve is determined on, the author recommends that a portion of the nerve should be removed; a point of practice that was very amply and luminously discussed in the Dissertation which gained the prize in 1813. Mr. Swan, in relation to this subject, says, "I think it is a question whether the nerves have the power of communicating their influence to other nerves whose communications with the brain have been cut off, in the same manner the arteries have whose direct communication with the main trunk has been intercepted by a ligature; but I think we may safely say, that, at all events, it can only exist in a trifling degree, and in some particular cases."

Inferences, the reverse of those drawn in the passage just cited, are satisfactorily established in the Dissertation which gained the prize in 1813. This is a point of very great importance in regard to practice: it is, therefore, discussed in the Dissertation just cited, with a degree of care and profundity worthy of the object. We shall not express what our feelings are on finding it noticed in so superficial a manner as it is by Mr. Swan, seven years subsequently to the publication of the discussion just alluded to. We shall have more to say on this subject hereafter.

The sixth chapter is "on Inflammation of Nerves." This chapter occupies but three pages, and does not present any thing that is novel and original. This, too, is a subject which is treated on in a very interesting manner in the Dissertation which gained the prize in 1813: and, in the course of the long and profound, yet luminous, investigation devoted to it, the author, whose name we have not mentioned, has every where scattered an abundance of hints, for practical views, of the most important character.

"Ulceration of Nerves," is the subject of the seventh chapter. No example is adduced of the exclusive ulceration of nerves; the author's observations on this point relate merely to ulcerations of nerves involved in ulcers of the adjacent structures. In one of the two cases related in this chapter, the most remarkable circumstances were a thickening of some of the nerves of a lower extremity in which there was an old ulcer on the tibia, whilst others were emaciated and enveloped in a peculiar sort of fat. Several varicose veins were observed in different parts of the sciatic nerve, and some of the nerves were unusually soft. The other case presents nothing particularly remarkable. A case from Morgagni, and another by Sir Everard Home, in which ulceration of nerves, of some considerable size, was supposed to exist, are referred to.

The eighth chapter is on "Tumors in the Nerves."—"When a tumor is forming in the substance of a nerve (the author says,) it causes very violent pain, which sometimes affects the whole nerve in which it is contained." That this statement is not correct as a general description, we have already shown, by quoting a case in which a tumor had formed in a nerve without the existence of pain. Mr. Swan relates a case, in which a tumor in a subcutaneous nerve formed about the middle of the leg, without any assignable external cause, and which was removed, with a favourable result, by excision. Cases by Portal, Dr. Denmark, Mr. Abernethy, and Sir Everard Home, are adduced in an abstract form; the whole of which, except that of Portal, (which is not an important one,) had been noticed in the Dissertation which gained the prize in 1813, and were there made the bases of numerous important inferences that have not been arrived at by Mr. Swan; whilst the same Dissertation presents all the views taken by this writer; differing from the latter only in being much more perspicuous and comprehensive, and infinitely more fertile in indications for the purposes of the practice of medicine.

After this, the author enters on a discussion of the question whether it is better merely to divide, or to remove a portion of the nerve, in certain cases, when its communication with its centre is to be interrupted. He says, "When a nerve has been divided, re-union in course of time generally becomes perfectly established, so that it performs its functions as well as if no division had ever taken place. When a portion of a nerve has been removed, and especially if it be a large portion, the breach is with the greatest difficulty, if ever, repaired, when it happens in the case of a nerve of the largest size."

The author of the Dissertation which gained the prize in 1813 had settled this point quite as well as the existing facts would permit it to be done; whilst he endeavoured to determine, precisely, the extent to which the reparation of nerves might be effected, and the time required for such a reparation. On this point Mr. Swan has added nothing to our knowledge; nor has he given any original hints for its improvement.

The ninth chapter is on "Injuries of the Nerves of Voluntary Motion, &c." It is so concise that we shall wholly transcribe it.

"The symptoms occasioned by injuries of the nerves are frequently very violent, but they are so various as to make it impossible to say what will be the result of an accident that has affected them; as sometimes an apparently trifling injury of them will bring on bad symptoms, whilst at another time a more violent one will not be attended by a single untoward symptom."

"The Treatment of Divided Nerves," is the subject of the tenth chapter. The author commences with stating, that "when a nerve has been divided, if the external wound is healed by the first intention, very little pain is felt in the nerve, in proof of which I shall relate the following case." The case shows that in one instance no pain ensued from division of a nerve of the thumb, when the union of the wound was effected by the first intention. The author has adduced no other than this single point of evidence, that is but *negative*, as the ground of a *general positive* inference. The rest of this chapter contains no pathological observations or therapeutical precepts—if we except the details of some not particularly remarkable cases; from which, however, the author has not drawn any original inference, excepting that above cited,—that are not presented, in a manner that precludes comparison, in the Dissertation which gained the prize in 1813. One of the cases is related, because it seems to prove that the sciatic nerve was wounded in a fracture of the neck of the femur below the capsular ligament, (an accident which the author thinks happens not unfrequently,) and because "the appearances of the limb in this case were different from what are usually presented in fractures of the neck of the thigh-bone; and were such that, without great care, might have been mistaken for a dislocation of the bone backwards."

The eleventh chapter is on "the Treatment of Punctures, or partial Divisions of Nerves." This chapter commences with a general account of the symptoms which occasionally ensue from the puncture or partial division of a nerve of any considerable size. The author then enters on an hypothetical discussion respecting the symptoms in question. This we shall wholly transcribe, not because it appears very luminous or satisfactory, but because we think it prudent to expose, in as complete a manner as possible, whatever is peculiar to the author in this Dissertation.

"When a nerve has been wholly divided, each portion of it immediately retracts, so that a considerable space is left between them. When only a partial division has taken place, the divided portions retract in the same manner, though not in so great a degree; leaving a space in the divided part of the nerve, whilst the undivided portion remains of the same length as before the division. Now each nerve, or at least the greatest part of them, is composed of different fasciculi, and these fasciculi, in most instances, communicate together; should one complete fasciculus be divided, that had not any communications with the others of which the nerve is composed, it would retract, and leave its fellows in the same state as before the division; and it is most probable that there would be no other difference, as from irritation, &c. than when the nerve is completely divided; but if a fasciculus is partially divided, or if it is wholly divided, and at the point of division it was connected with the adjoining fasciculus, the retraction of the divided parts would stretch those that were joined to it, and thereby cause considerable pain; for we know that this stretching of a nerve produces violent symptoms, as in cases of tumor. I will endeavour to explain my meaning by the following:

1. Undivided portion of nerve.

2. Two divided portions.

3. Two fibrils by which the divided and undivided portions communicated, and which, by the retraction of the divided portions, must be kept on the stretch.

"But again, should a nerve be wholly divided, except one fasciculus, and at this place there were not many communications, the great retraction of the divided parts would very much keep on the stretch the undivided fasciculus. Any one may be satisfied of this, by taking an animal soon after it is killed, and laying bare a nerve, and almost entirely dividing it: the divided portions will be seen to have retracted in some degree; but, immediately on dividing the remaining part, each end of the nerve will retract in the quickest possible manner to a much greater distance than it did before the undivided part was cut through; thereby clearly proving that, as there was nothing but this small portion to prevent the retraction, it must have been kept very much on the stretch."

After this, the author remarks that, from his experiments in partially dividing the nerves of animals, it does not appear to him that more suffering ensues from such an injury, in the inferior animals, than from the entire division of a nerve: a fact which is somewhat adverse to his explanation; and he is, hence, induced to suppose that there are some peculiarities in the nervous system of man that give origin to such a diversity of results; or that there must be some peculiarities in the constitution of those persons in whom the severe morbid consequences have taken place; or that it is only when a punctured nerve becomes inflamed that those same effects happen. To all those suppositions, excepting the last one, there can be no objections; but, with respect to the last, it is satisfactorily shown, in the Dissertation which gained the prize in 1813, that this inflammation of the nerve that is the seat of the severe morbid symptoms, cannot reasonably be regarded as the *cause* of the symptoms; and must itself be only a consequence of some other inappreciable condition.

This remark will apply to the evidence developed by anatomical researches, in a very extensive manner; it has recently been very forcibly presented to us in respect to the whooping-cough: we were beginning to hope, from some appearances witnessed on dissection, that we had discovered the cause of the phenomena peculiar to this disease, and especially of the obvious affection of the nervous system which so frequently arises in its progress, (and which seems to have happened with particular frequency during the epidemical prevalence of the disease in London, in the last winter;) but these hopes were unfortunately dissipated, by our finding precisely similar appearances (as far as our senses inform us,) in the body of a child who never was affected with the whooping-cough.

Mr. Swan adduces (from his own observations,) histories of some cases in which severe symptoms, attributable to wounds of nerves, ensued from the operation of venesection; but they present nothing extraordinary: some others, more or less interesting, are cited from the works of SABATIER and LARREY. His therapeutical precepts are only repetitions, not remarkable for any extraordinary degree of precision respecting their application, (which might render repetitions of general precepts of some value,) of what has been advanced by the ordinary writers on this subject; whilst several very interesting facts bearing on this point, contained in the Dissertation which gained the prize in 1813, and proper to that work, are not noticed by him. Mr. Swan then treats of Tetanus. He first describes its symptoms, and afterwards adduces the following remarks respecting its etiology.

"The cause of the disease is a violent irritation of the nerves, produced either by the suppression of perspiration, as when it comes on from cold, or from an irritation of the nerves of a wound, either where the large nerves have been injured, or where their more minute branches are irritated from the unhealthy action in the wound. Larrey* relates three cases where it was produced by an injury of the larger nerves. In the first, the anterior crural and sciatic nerves had been injured by a ball; in the second, the median nerve had been tied with the brachial artery; and in the third, the nerves had been tied in amputation of the leg.

"Some have supposed that this complaint proceeds from some disease in the parts about the medulla spinalis. The changes from the healthy appearance have been found in the membranes of the medulla spinalis in some cases of this kind, there can be no doubt; but whether they are the consequences of the violent contractions of the muscles or accidental occurrences, cannot, I think, be determined: at

* *Memoires de Chirurgie Militaire,* tom. iii. p. 290.

all events, I should hardly be inclined to think that the changes in these parts can have been the causes of the disease."

With respect to the doubts expressed in the last sentence: they had been previously stated in the Dissertation which gained the prize in 1813, and connected with a close investigation and comprehensive inferential disquisition on this subject. Dr. Copland has mentioned to us that he has, in one instance, (the only research of the kind he has yet made,) found the spinal marrow and its membranes highly injected with blood, in a hare which had been hunted just previously to its death; an observation which, though it be solitary, is qualified to throw much doubt on the propriety of regarding the inflammatory appearances found about the spine as the *cause* of tetanus. Dr. Saunders, of Edinburgh, says,

"1. If any muscle, voluntary or involuntary, is affected with spasm, and during this affection the person dies, on examination it is found that the nerves which supply the spasmed muscle are covered with turgid red vessels at their visible origins, or where they appear to set off from the brain, medulla oblongata, or spinal marrow.

"This turgescence, and the effects of turgescence, are in the ratio of the degree and duration conjointly of the spasm or convulsion.

"The turgid vessels, in every obstinate and severe case, may be traced into the substance of the spinal marrow, by the sides of the striæ, which seem to be the continuations of the nervous filaments; as also along the nervous cords, through their sheaths formed in the dura mater.

"The position of the body after death has no appreciable influence on these appearances: they are observed anteriorly or posteriorly, in the loins, thorax, cervix, or within the skull, bearing strict relation to the parts which have evinced spasmodic action.

"But the nerves serving the muscles which have not laboured under spasm or convulsion, are free from turgid vessels.

"2. If the tetanic affection is confined to the jaw, certain nerves arising from the tuber annulare and medulla oblongata, are found in the state above described.

"But if the tetanic affection involves the whole inferior extremities and the trunk of the body, as well as the jaws, then the origins of the nerves, from the tuber anulare to the cauda equina, are covered with turgid red vessels.

"In short, the nerves exhibiting such turgescence at their origins correspond, in number and situation, with the muscles which have exhibited inordinate contraction. I have conducted this investigation for about sixteen years, and have not met with one exception.

"Some are of opinion, that I maintain that the spinal marrow, its nerves and membranes, are always affected with turgid vessels in tetanus: this is incorrect. I have examined cases of trismus, in which the spinal marrow, its membranes and nerves, were almost entirely sound, from the atlas to the lumbar vertebræ. In these instances, however, not only the origins of the nerves at the medulla oblongata, but the medulla itself, was inclosed with a close net-work of turgid red vessels.

"There are many other appearances within the cranium and spinal canal, more or less connected with spasms and convulsions; but those which I have here related are uniform, and accordingly constitute, we have reason to believe, an essential part of these diseases.

"Morbid changes in the organization, as of substance of the brain and spinal marrow, or in their envelops, belong to another order of maladies, and, when present in those affected with spasms, the symptoms always indicate complication."

The very interesting nature of this subject has led us to digress more from an analytic examination of the work of Mr. Swan than we intended: but our readers, we do not doubt, will wish that we had endeavoured, on other points, to render this article more interesting than it must be, if constituted solely of an abstract of the (almost without exception) very imperfect and common-place discussions of the author of the Dissertation before us. We proceed in our analysis according to the mode we have adopted in the former part of it; and the next remark we have to make is, that Mr. Swan's observations respecting the treatment of tetanus present nothing that is original, and are of the most common-place character.

This brings us to the twelfth chapter, which treats of "the Effects of Ligatures on Nerves." It begins with the assertion, that "many experiments have been made on animals, to show the effects which a ligature applied on a nerve has on the parts to which it is distributed; but they do not show much respecting the changes the nerve itself undergoes, or the diseases the ligature might occasion."

The Dissertation which gained the prize in 1813, presents the results of a series of experiments instituted for the express purpose of ascertaining the effects of ligatures on nerves: the object is a practical one, and, like all objects of this kind, it is discussed with a relative view in the Dissertation just cited. The common-place remarks of Mr. Swan, and the few observations he cites from other authors, will but ill supply the place of the discussion just referred to, for the purposes of the medical practitioner.

The thirteenth chapter is on "the Compression of Nerves." We shall give an abstract of the several cases which Mr. Swan introduces on this subject; because it is true that they present some *new observations*, and our opinions respecting the value of these observations may differ from that of many other persons. The first two cases are cited from Richerand, to show that, when a certain degree of pressure is continued for a certain length of time on the trunk of a nerve, the parts to which such a nerve is distributed may be deprived of their powers of sense and motion.

"A young man went to sleep with his head resting on his arm, the outside of which was placed on the edge of a table so as to compress the radial nerve; and the consequence was an insensibility of part of the integuments, and a paralysis of the muscles at the back part of the fore-arm. These symptoms were removed by irritating frictions in the course of the nerve.

"Compression of the median nerve during an operation that was performed on the fore-arm, produced a numbness of the limb; the sensibility was not restored before the end of forty-eight hours."

Mr. Swan then says, that, when the nerves have been injured from a continued pressure, "the best remedy will be frequent frictions of the hand, and the use of a stimulating embrocation;" for which he gives the following recipe:—"℞. Linim. sapon. comp. ℥x; Liquoris ammoniæ, ℥ij. M." After citing a case from Portal, the author relates one to show that the bladder, when suffered to become distended in paraplegia, will tend, by its pressure on the nerves going to the lower extremities, "very materially to retard, if not to prevent, their restoration." Another case is then cited from Portal, to prove that "sometimes the nerves suffer so much from a sudden compression as to lose entirely their power, which they have the greatest difficulty in recovering." That "the same accident happens sometimes to the nerves of the axillary plexus, from an injury of the shoulder," is shown by a case narrated by the author. He then remarks that—

"A nerve may be extended some way without giving pain or uneasiness, as I have frequently observed in making experiments, when I have passed a probe under the sciatic nerve, and drawn it from its situation; and as is shown in cases of popliteal aneurism, when the swelling may get to some size before much pain is produced.

"But when a nerve is extended in any considerable degree, pain is excited; and, if the extension is increased, the pain is increased in proportion, till at length the nerve begins to ulcerate, and, if the pressure is not removed, is almost entirely destroyed."

After this Mr. Swan, in continuation, remarks, that "violent blows on the back sometimes cause bad symptoms, though they are unattended by much apparent external injury;" and a case is cited in proof. He then relates a case, "because it shows that, after an injury of the medulla spinalis, the nerves may be sufficiently restored to be capable of performing their functions so as to produce feeling, when they are not so, in the least degree, for the production of voluntary motion; and likewise because it shows that, when the medulla has not been too much injured, if every compressing power is removed, a very great degree of restoration may be effected." With this case the thirteenth chapter terminates.

The fourteenth chapter presents the results of "an experimental inquiry into the process nature employs for repairing Wounds of Nerves." The author says,

"Many experiments have been made by physiologists, to prove that, when a nerve is divided, all sensation and motion are lost in the parts to which it was distributed; and that, after the re-union of the divided parts, it performs its functions as well as before the division. I had always understood that this was a point generally agreed upon by physiologists; and it has been so well illustrated, especially by the experiments of Dr. Haighton, that it is difficult to conceive how, after an elucidation so satisfactory, any doubt should remain on the question. But, when we find it contradicted by several eminent men, so much hesitation is produced in the minds of those who are unbiassed by any favourite hypothesis, as to lead them to make an experimental inquiry into the subject for themselves."

After noticing the impertinent expressions of doubt of Richerand on this subject, and the manifestations of ignorance respecting it, of Delpech, Mr. Swan says,

"Amidst these contradictions, as I was not aware that any experiments had been instituted to show the process nature adopts for the restoration of the parts, and as I could not obtain from books knowledge sufficiently satisfactory, I have made the following experiments, which I trust will account for many things respecting injured nerves, which surgeons do not at present seem clear about."

We can discern nothing of any value in the results of the experiments of Mr. Swan, that is not presented in the Dissertation which gained the prize in 1813. The general results of observations at several diverse periods, from various species of wounds of nerves, were stated in that Dissertation. Mr. Swan has shown the results of observations at a *greater number of periods;* but whether they are of importance, is a subject of mere opinion, and may be doubted until this importance is proved. The author of the earliest dissertation of the two on this subject, of course, thought that observations of the appearances at a certain number and diversity of periods, were sufficient for every useful purpose, or he would have extended his experiments; or else he must have considered that the results would not be likely to be sufficiently useful to warrant the additional torture they would cause to animals which, by their destinies, are submitted to our power. The observations of Mr. Swan do not appear to us to be calculated to make that author alter such an opinion, should he have entertained it: Mr. Swan does not, we repeat, prove the importance of his additional experiments, by any precise inferences, in the "conclusions" from them, with which his dissertation terminates.

Notwithstanding the weariness of the reader who has accompanied us so far in this article, we have yet a few remarks to offer before we part with him on this occasion. Mr. Swan, in the motto to his Dissertation, says, "Non scribo hoc temere. Quo minus *familiaris* sum, hoc sum ad investigandum *curiosior:*"—an assertion which but ill accords with his neglect of the Dissertation on the same subject that gained the Jacksonian prize in 1813.* We must suppose that he has not perused that Dissertation; for (without considering that several inferences are satisfactorily established in it that are adverse to the notions of Mr. Swan,) we cannot suppose that, had he been acquainted with the Dissertation alluded to, he would have ventured to publish what he must then have been conscious contains so little of valuable matter that is not to be found in the one which had preceded it; and what presents also, in every respect, so very inferior a view of the subject referred to. We repeat, in a general allusion, what we have said in relation to every separate chapter in the Dissertation of Mr. Swan, that, excepting a very few facts, which we consider to be of but trivial importance,—and which the author has not made to appear otherwise, by any original inferences from them,—that it contains no original information of at all considerable utility to medical practitioners. In making this assertion, we do not, with only two or three instances of exception, (as the notions about the connexion of the facial nerves with the auditory faculty; those respecting the reason why sensation is lost whilst the power of motion is preserved, and the converse [which are not novel]; and some observations on the structure of the pituitary membrane in the nostrils of the horse, which are original;) allude to what is to be found dispersed in numerous and foreign authors; we state it in reference to the Dissertation, on the same subject, that gained the Jacksonian prize in 1813. We are confident that every reader will be satisfied of the propriety of this assertion, on making a comparison of the two works. We have not cited the passages in proof of our statement from the earlier one, because this Dissertation is constituted of such an intimately-connected series of inferences and indications, or is, in other terms, such an admirable illustration of the motto to it,† that hardly any passages could be extracted in an insulated manner, without doing injustice to the author, by presenting a very imperfect view of his disquisitions on al-

* This Dissertation, it may be proper to remark, was printed and published, by Callow, in 1815.

† Namque aliud ex alio clarescet; nec tibi cæca

Nox iter eripiet, quin ultima naturaï

Pervideas; ita res accendunt lumina rebus.

most any point considered in them. The reader will here, probably, be disposed to inquire how it has happened, then, that the prize has been assigned for this Dissertation of Mr. Swan: we are perplexed for a plausible conjecture in reply. The most probable one we can form is, that the members of the College who examined the memoirs (if there were more than one,) considered themselves obliged to award the prize to the *best* of them, whatever it might be. But this explanation almost precludes a supposition that more than one memoir was presented to them; for it is hardly possible to conceive that a dissertation so devoid of value as one from which it is impossible to select any thing of at all considerable interest or importance (with the two or three exceptions above stated,) that cannot be pointed out (expressed in at least as perspicuous and satisfactory a manner,) in *one book alone*, and that an English one, which was published five years since; it is not reasonable to suppose that such a dissertation could have been regarded as the *best*, had there been any contest for the decision. We must not express a doubt of the Court of Examiners being well acquainted with the Dissertation which gained the prize in 1813.

We have yet to observe, that there are three plates attached to this Dissertation: one, to show the figures and distribution of some regenerated nerves in a rabbit's leg; another, to represent the figures and distribution of the sinuses in the pituitary membrane of the nostrils of the horse; and the third, to show the situation and distribution of the more superficial nerves about the face and neck, especially those most frequently affected in tic douloureux. The preparation (made by Mr. Swan,) according to which the last plate was executed, is preserved in the museum of the College of Surgeons. So minute a representation of those nerves, is a thing of no small value; and we have regarded it with much gratification,—not but that we have seen as minute and intricate preparations, as well as figures, before,—but, unless one knows by whom they were executed, no confidence can be felt of their exactness. Such preparations, it is true, are preserved in museums at public schools, and sometimes with the names of eminent professors attached to them, though they have been the production of their pupils; and few young men can resist making attempts to improve, artificially, the appearance of their work, or to remedy, in the same way, the effects of an unlucky stroke of the scalpel, destroying the results of many days', or even weeks', labour. Hence it is that, in many such preparations, our admiration is conferred on the intricate distribution of varnished and painted strings of cotton and catgut.

## Figures and Tables

**Figure f1:**